# Optimization of simultaneous adsorption of nickel, copper, cadmium and zinc from sulfuric solutions using weakly acidic resins

**DOI:** 10.1038/s41598-024-58366-3

**Published:** 2024-03-29

**Authors:** Somayeh Kolbadinejad, Ahad Ghaemi

**Affiliations:** School of Chemical, Petroleum and Gas Engineering, Iran University of Science and Technology, Tehran, Iran

**Keywords:** Metal ion, Resin, Adsorption, Kinetic, Thermodynamic, Response surface methodology, Environmental chemistry, Chemical engineering

## Abstract

In this research, the adsorption of nickel (Ni), copper (Cu), cadmium (Cd), and zinc (Zn) from real sulfuric leaching solution with weakly acidic resins has been studied using response surface methodology (RSM). The adsorption process on two weakly acidic resins has been investigated as a function of pH, time, temperature, and resin dosage. The experimental results indicate that the amino phosphoric acid resin removed Ni, Cu, Cd, and Zn from an acidic solution very efficiently. Based on the central composite design (CCD) on the RSM, the statistical criteria of correlation coefficient (R^2^) values of Ni, Cu, Cd, and Zn are 0.9418, 0.9753, 0.9657, and 0.9189, respectively. The adsorption process followed the pseudo-second-order kinetic model and the thermodynamic calculations indicated the chemical interaction between the resin surface and the metal ions. Enthalpy values greater than zero indicate that the adsorption reaction of the metals is endothermic. The optimal adsorption process was carried out at time of 20 min, temperature of 30 ^0^C, pH of 5, and resin dosage of 4 g/L. In these conditions, the adsorption capacity of nickel, copper, cadmium, and zinc were obtained 13.408, 7.087, 4.357, and 15.040 mg/g, respectively.

## Introduction

Increasing the request of valuable metals such as Ni, Cu, Cd and Zn in various industries and decreasing the primary sources of these metals in nature are the reason for recovering these metals from industrial waste^1^. Ni is a hard and corrosion-resistant metal and is used in catalyst production, battery, electrical industries^2,3^. Cu, like silver and gold, exists in natural state without combining with elements^4,5^, and due to its electrical conductivity, high workability, and corrosion resistance is used in electronics, military, shipbuilding, construction, and industrial equipment^6^. Cd is harmless to human health up to 0.53 mg/kg, but more than this amount, Cd is a heavy and toxic metal^7,8^ that from melting and extracting metals enters to the environment^9^. Zn, despite being useful for human health, causes poisoning in excess of the permissible limit^10^. The filter cakes of the Zn industry contain Ni, Cu, Cd, Zn and etc. which can be considered as a secondary source for the supply of these metals, therefore, the recovery of precious metals is needed and includes leaching and separation steps^11,12^. In the leaching stage, there are heterogeneous reactions between the solid phase and the liquid phase and the solid phase is dissolved^13^, but dissolution is not selective and requires the selective separation of desired metals in the next steps^14,15^. The recovery of metal ions from the leaching solution depends on the pH, composition, concentration of ions in the solution. Among processes, it can mention sedimentation^16^, electrowinning^17^, solvent extraction^18^ and ion exchange resin^19^. In order to reduce industrial costs, resins are used for some reasons such as: in the reversible process of using cationic/anionic resins, the ion exchange between the cation/anion in the functional group of the resin occurs with the cation/anion in the solution, respectively. Equation (1) shows the ion exchange reaction for cationic resins^20,21^:

1 $$ R^{ - } A^{ + } + M^{ + } \leftrightarrow R^{ - } M^{ + } + A^{ + } $$

Functional groups give properties such as acid, alkali or chelation to resins. There are four main types: strong acid, strong alkali, weak acid and weak alkali, whose functional groups are different^22^. Fixed bed columns are used for industrial applications^20^ and adsorption selectivity is related to the metal complex at any pH^22,23^. Sufficient residence time is one of the important factors to increase adsorption efficiency. If the flow intensity increases, adsorption efficiency decreases due to the reduction of required time^24^. Adsorbents are classified into: carbonaceous materials, industrial and agricultural wastes, polymer adsorbents, mineral materials, bio adsorbents, composites and adsorbents with special structure^25^.

Based on the studies, some resins such as: amberlite IRC 748^23^, Pyrolite S 930^26,27^, Lewatit Mono-Plus TP 207 XL^28^, NDC 984^29^, activated carbon prepared from sewage sludge^30^ nano graphite^31^ and various nanocomposites^32^ are more selective to the separation of Ni from Cd, Cu and another metal ions. Results of comparisons between Amberlite IRC 748, Unac SR 5, Prolite S 930 and Dowex M 4195 resins for adsorption of Ni and Co from high pressure acid leaching solution, show more nickel is adsorbed by Dowex M 4195 than the other resins^33^. NPG@Fe3O4 nano-absorbent was produced with mixing of graphene nanoparticles and Fe_2_O_3_ and Fe_3_O_4_, which allows easier separation of Ni from aqueous solutions^34^. D 401 chelate resin^24^, S930 chelate resin^27,35^, resin derived from acrylonitrile-divinylbenzene copolymers^36^, Dowex M4195^37,38^, Levatite TP 220^38,39^ and WRAM^15^, polyglycidyl methacrylate-glycine porous chelating resin^40^, resin with group of polyhydroxamic acid—polyamidoxime^41^, chelating group of amidoxime^42^, Dowex XUS 43578^43^, cross-linked magnetic chitosan^44^, Magnetite Nano-Adsorbent (MNA)^45^, magnetic chitosan beads (MCSB)^46^, sawdust chitosan nanocomposite beads^47^, melamine-diethylene triamine pentaacetic acid resin^48^, nanocomposite powder activated with biological materials^47^, granular activated carbon produced from palm kernel shell^49^, coal fly ash^50^, bio adsorbent^51^, activated carbon prepared from sugarcane papyrus^52^, Zeolite-Based Geopolymer adsorbents^53^, a novel nanocomposite adsorbent, graphene oxide modified with magnetite nanoparticles and Lauric acid containing ethylenediaminetetraacetic acid (GFLE)^54^, Hydroxy propyl picolyl amine (HPPA)^55^ are useful for selective recovery of Cu, Zn and Cd. The several resins including Dowex M 4195 (bis-picolylamine), Amberlite IRC 748, Prolite S 930 (iminodiacetate) and Prolite S 991 (amine/carboxylic) are more selective for Cu in the solution containing Ni, cobalt, iron, Zn, manganese and aluminium^56^. Adsorption of Cu in acidic streams was studied using polymeric resins including Levatite Monoplas TP220, Levatite Monoplas SR7, Levatite AF5, Prolite A 830, Prolite S 984, Prolite A 40 TL and Dox PSR 2. The adsorption efficiency of Cu was the highest using TP220 Monoplas Lavatite^57^. The adsorption behavior of Lewatit MonoPlus TP220 for Cu in the presence of ABSNa50 surfactant was better than other adsorbents (adsorption capacity ≈ 10 mg/g)^58^. Polymeric adsorbent of Amberlite XAD7HP used for As, Cd and Pb metallic ions recovery^59^. A new chelating resin with chemical modification of styrene–divinylbenzene copolymer adsorbed toxic metal ions from aqueous environments^60^. The adsorption of Cd, Ni, Cu and Pb from the aqueous solution has occurred with amberlite IRA 402 and amberlite XAD7HP that were functionalized by chelating agent Direct red 23 (DR 23). The adsorption of Cd was higher than other ions^61^. A summary of the operating conditions and functional groups of the industrial resins is given in Table 1.

**Table 1:** Operating conditions and functional groups of the industrial resins.

Resin type	Functional groups	Operating conditions				Reference
		Time (h)	Temperature (°C)	pH	S/L (g/l)	
Amberlite IRC 748	–CH_2_N–(CH_2_COOH)_2_	–	25	5	2.5	^23^
Pyrolite S 930	–N(CH_2_COOH)_2_	6	20	2.5	1:10	^26^
Lewatit Mono-Plus TP 207 XL	NH(OOH)_2_	24	25	–	–	^28^
Activated carbon prepared from sewage sludge	Carbon-Based materials	1.5	55	8	4	^30^
Nano Particles of Graphon (NPG)@Fe3O4	Carbon-Based materials	0.5	25	9	–	^34^
Dowex M4195	Bis(2-pyridylmethyl) amine	3.5	50	–	–	^37^
Magnetite Nano Adsorbents (MNA)	Magnetic-Based materials	–	25	5.4	0.05	^45^
Glutaraldehyde Cross-Linked Magnetic Chitosan	Magnetic-Based materials	6	28	5	1.5	^46^
Sawdust Chitosan Nanocomposite		1.1	30	5–6	0.05	^47^
Dealumination of Coal Fly Ash	Coal Fly Ash	6	25	8	–	^50^
Activated carbon prepared from sugarcane papyrus	Carbon-Based materials	1	25	6	30	^52^
Zeolite-Based Geopolymer adsorbents	Zeolite-Based materials	2	25	–	2	^53^
Nano composite adsorbents	Graphene oxide/ Lauric acid + Ethylenediaminetetraacetic acid nanoparticles (GFLE)	1.45	40	1	0.28	^54^
XUS43605	Hydroxy propyl picolyl amine (HPPA)	2	25	2	–	^55^
Lewatit Mono-Plus TP 220	Bis(2-pyridylmethyl) amine	24	25	–	–	^58^

Polymeric resins have advantages such as non-volatile, chemical stability, insoluble in water and can be reused in subsequent surface adsorption cycles. A "weak" acidic resin will only ionize within a limited pH range. While a "strong" acidic resin shows no change in ion exchange capacity with pH changes. The ion exchange capacity of weakly acidic resin depends on pH value of solution. Also, weakly acidic resins can gain or lose protons with buffer pH changes. Regeneration and recharge provide another feature of selectivity for these types of resins.

Nowadays, response surface methodology (RSM) is one of modeling and optimization method that is used for understanding the behavior of systems in chemical processes and optimizing their performance^62,63^. RSM reduces systematic errors by estimation of investigational error and also requires fewer computer simulations with fewer experiments^64^.

It is noted that the recovery of heavy metals as a secondary resource is still an evolving research area and there is scope for optimization and integration of new and/or existing technologies.

The innovation of the current research is the optimization of the simultaneous adsorption conditions of metals including Ni, Cu, Zn and Cd from the pregnant leaching solution obtained from the low-grade filter cake. Two weakly acidic resins including CH020 and CH030 have been used for adsorption of the metals from the leaching solution. The RSM model was used for experimental modeling and optimization of the process. The kinetic and thermodynamic adsorption models have been investigated to identify the resins behavior. The effect of operating parameters including temperature, pH, time and the resin dosage on adsorption rate of Ni, Cu, Zn and Cd were evaluated.

## Material and methods

### Materials

In the experiments, the leach solution was obtained by leaching of low-grade filter cake from Zn factory. The leaching solution was prepared at solid–liquid ratio of 0.09 g/mL, particle size of 177 mic and of pH 1.5. According to the leaching solution, it is tried to separate Ni, Cu, Zn and Cd by surface adsorption with resins by commercial titles of CH020, CH030, C100, and C100E from Canftech Company. The solution pH was preserved by sodium hydroxide for a range between pH 2 to 10. Chemicals like sulfuric acid 98%, distilled water, and sodium chloride have been bought from Merck.

### Methods

The RSM based on CCD approach is a set of statistical technique for experimental modelling of the process. Also, RSM was used for optimizing the Ni, Cu, Cd and Zn adsorption affected by several independent variables and reducing the number of experiments^65–67^. The quadratic polynomial for predicting the optimal effects of effective parameters is expressed in Eq. (2).

2 $$ y = \beta_{0} + \mathop \sum \limits_{i = 1} \beta_{i} X_{i} + \mathop \sum \limits_{i = 1} \beta_{ij} X_{i}^{2} + \mathop \sum \limits_{i = 1} \mathop \sum \limits_{j = i + 1} \beta_{ij} X_{i} X_{j} + \varepsilon $$

where y is the predicted response, β_0_ is the offset term, X_i_ and X_j_ are the independent variables, β_ii_ and β_ij_ are the interaction coefficients, respectively. ε is an unpredicted parameter that is determined experimentally. The aqueous samples were analyzed by flame atomic absorption spectroscopy (AAS/ model: WFX-220B) with Eq. (3), the adsorption capacity of resin can be calculated as follow:

3 $$ q_{e} = \frac{{\left( {C_{i} - C_{e} } \right)V}}{m} $$

where q_e_ is the adsorption capacity (mg/g), where C_i_ and C_f_ are the initial and final ion concentrations, respectively, m is the mass of used resin (g) and V is the solution volume (L^−1^). The adsorption percentage of each metal ion is determined by Eq. (4):

4 $$ Adsorption \;\left( \% \right) = \frac{{C_{i} - C_{f} }}{{C_{i} }} \times 100 $$

The statistical criteria of correlation coefficient (R^2^) was used to evaluate the accuracy and performance of the model, and the accuracy between the predicted values and the actual values^68^. R^2 ^was calculated as follow:

5 $$ R^{2} = \mathop \sum \limits_{i = 1}^{n} \left( {X_{predicted} - X_{actual} } \right)^{2} /\left( {X_{predicted} - X_{mean} } \right)^{2} $$

where X_actual_ and X_predicted_ are the experimental and the predicted values by RSM, respectively. X_mean_ is mean value of data and n is the number of data points.

Temperature, pH, resin dosage and time are independent variables. The combination of two resins was also considered as an effective factor. Alpha is the distance of each point from the center in a centered composite design. Points in the cube represent alpha values less than one, values on the faces of the cube represent alpha equal to one, and points outside the cube represent alpha greater than one. Here, alpha is chosen equal to 2 and the values between the minimum and maximum of each parameter are divided into 5 parts. Design experiment software proposed 60 experiments that their coding and actual values are given in Table 2.

**Table 2:** Independent numerical variables with the actual and coded levels.

Independent variables	Unit	Coded variable	Coded variable				
			− 2 ${\varvec{\alpha}}$	− 1 ${\varvec{\alpha}}$	0	+ 1 ${\varvec{\alpha}}$	+ 2 ${\varvec{\alpha}}$
Time	min	A	5	10	15	20	25
Temperature	C	B	25	30	35	40	45
pH	–	C	2.0	3.0	4.0	5.0	6.0
Resin concentration	mg/10cc	D	20	40	60	80	100

### Adsorption experiments

To start the experiments, it is necessary to prepare an acid leaching solution containing Ni, Cu, Cd and Zn ions. The leach solution was obtained by dissolving 13.5 g of low-grade filter cake with size of 177 microns in 150 cc of water and sulfuric acid solution with pH of 2.5 at 40 ^0^C for 1.5 h. In the next step, preliminary tests were performed to determine the range of important and effective parameters using CH020, CH030, C100 and C100E resins. According to the obtained results, two resins: CH020 and CH030 and the parameters of temperature, time and pH, and resin dosage were selected to continue the experiments. Based on the RSM, 60 experiments were performed in different conditions. In this way, 10 ml of leaching solution with a certain pH was placed as a feed in a special container with a 200-rpm stirrer in a water bath to adjust and stabilize the temperature. After reaching the desired temperature, the dosage of resin was added to the solution. After the required time, the sample was separated using filter paper. The initial and final concentrations of Ni, Cu, Zn and Cd in the leaching and adsorption solution were measured by flame atomic absorption spectroscopy (AAS/ model: WFX-220B). After every 60 samples, three standard solutions of metal ion were run to confirm the reliability of the results by the AAS. All experiments were performed in triplicate to determine their reproducibility, and the average concentration was determined using the mean and standard deviation (± SD).

The resins scanning electron microscopy-energy dispersive Xray spectroscopy (SEM, EDX) and X-ray diffraction (XRD) was done to identify physical and chemical properties.

Canftech CH020 and CH030 are a sort of chelating resin which have weakly acidic iminodiacetic acid [-CH_2_N-(CH_2_OOH)_2_] and weakly acidic amino phosphonic [-CH_2_NCHH_2_PO_3_-], respectively, in the styrene and divinylbenzene (DVB) copolymer with special microporous structure. The crosslinked styrene–divinylbenzene copolymer forms the structure of ion exchange resins. The interaction of CH020 and CH030 functional groups with metal cations are provided in Eqs. (6) and (7), respectively.

6 $$ \left[ { - {\text{CH}}_{2} {\text{N}} - \left( {{\text{CH}}_{2} {\text{OOH}}} \right)_{2} } \right] + {\text{M}}^{2 + } \to \left[ { - {\text{CH}}_{2} {\text{N}} - \left( {{\text{CH}}_{2} {\text{OO}}} \right)_{2} {\text{M}}} \right] + 2{\text{H}}^{ + } $$

7 $$ \left[ { - {\text{CH}}_{2} {\text{NCHH}}_{2} {\text{PO}}_{3} - } \right]^{2 - } + {\text{M}}^{2 + } \to \left[ { - {\text{CH}}_{2} {\text{NCHH}}_{2} {\text{PO}}_{3} {\text{M}}} \right] $$

### Resin regeneration

For regeneration, 10 cc of 20% (w) of sulfuric acid was added to the resin and stirred for 5 min at room temperature with a stirrer at 200 rpm. Then, the final concentration of metal ions in the aqueous phase was measured by flame atomic absorption spectroscopy (AAS/ model: WFX-220B) with acetylene–air flame atomization. The regenerated resin was reused in the adsorption cycle. This cycle was repeated up to 5 times with the same amount of adsorption. The regeneration results showed that the resins can be used several times without loss of absorption capacity.

## Results and discussion

### Resin characterization

**Figure 1:**
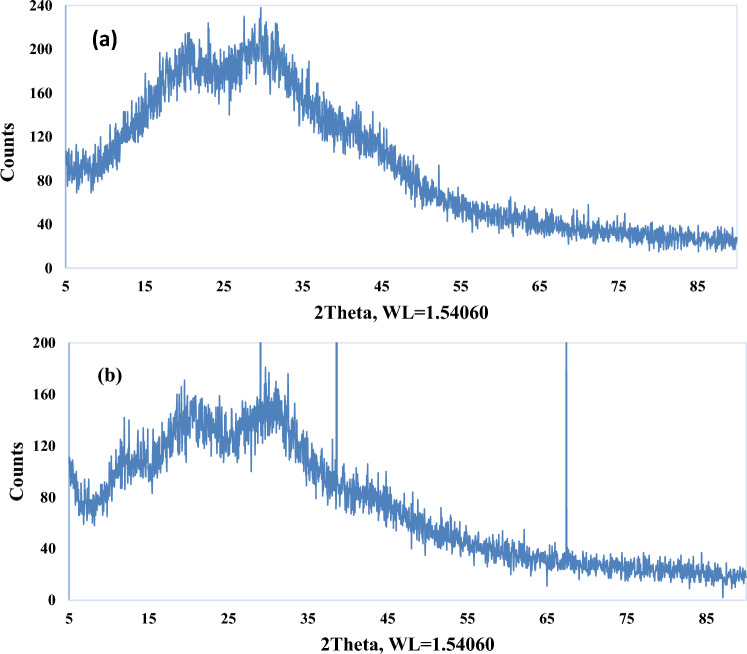
X-Ray diffraction analysis (XRD) of resins (**a**) CH020 and (**b**) CH030.

The formed materials and the crystallographic structure had been characterized by X-Ray diffraction analysis (XRD). The higher intensity of the resulting peak shows the higher crystallinity percentage of the sample and the more regular arrangement of the polymer chains. But the more irregular arrangement of the chains indicates by the wider resulting peak. According to Fig. 1a, b, the arrangement of the polymer chains in the two resins is regular and there is more opportunity and possibility of ion exchange.

**Figure 2:**
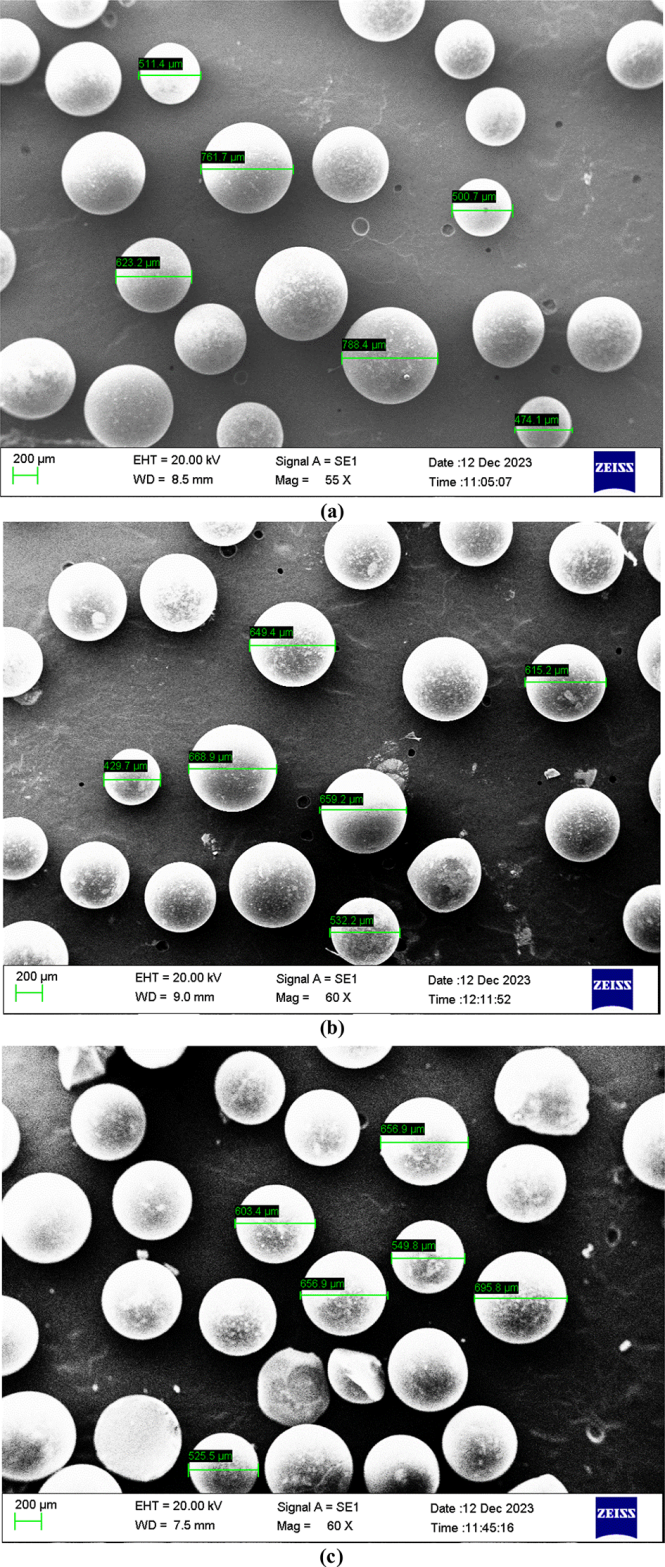
The scanning electron microscope (SEM) of resins before ion exchange: (**a**) CH020, (**b**) CH030 and after ion exchange, (**c**) CH030.

The scanning electron microscope (SEM) uses a focused beam of high-energy electrons to generate a variety of signals at the surface of solid specimens for analysis of the surface morphology of the resin before and after adsorption. Images of resins CH030 and CH020 by SEM are shown uniform microporous structure on the regular spheres in Fig. 2. Polymer particles have diameters in the range of 550–600 μm. After the ion exchange process, in the surface morphology of resin can be seen a small difference in the form of roughness. These changes are not much due to the structure of the resin, and for this reason, the resin can be regenerated and reused.

### Effect of operating condition on adsorption capacity

Adsorption capacity of metal from the liquid phase depends on several physicochemical factors including pH of solution, resin type and dosage, temperature, time, initial metal concentration and presence of other cations. In the experiments, an acid leaching solution of low-grade waste containing various metal ions is used for the metals removal. The effect of time, temperature, pH and resin dosage on adsorption capacity are investigated.

**Figure 3:**
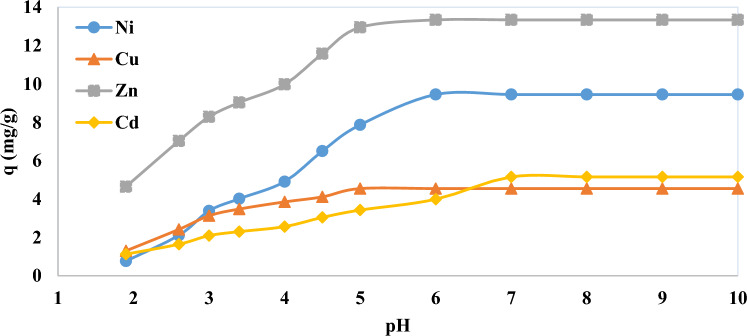
Effect of pH on the adsorption of Ni, Cu, Cd and Zn (mg/g) from aqueous solution by CH030 (time 30 min, room temperature, 5 g/L of resin).

pH is one of the most important and crucial parameters in the adsorption of Ni, Cu, Cd and Zn. Metal precipitation, ionization degree and activity of the resin functional group are considered in the pH adjustment^76^. Changes in the pH level and the presence of hydrogen ions in the solution affect the rate of ion exchange^77,78^. The experiments were done with 5 g/L of CH030 in the leaching solution at room temperature for 30 min in the pH range of 2–10. According to Fig. 3, the changes in initial pH are very extensive due to the effects of pH on the structure of the resin. At pH between 2 and 4 due to the increase of H^+^ and its competition with metal ions for resin sites, electrostatic repulsion occurs with the resin and smaller amounts of adsorption are observed. But at pH range of 4–6, a significant increase in adsorption values is observed due to the reduction of positive ions on the surface of the resin and reached a peak at pH 5–6 and then slightly constant. In pH of 6–10, metal ions can generally be precipitated as a hydroxide in solute^79,80^. With the increase of pH and increasing of OH^-^, metal cations have a greater tendency to form hydroxide deposits and sediments instead of adsorption, and real adsorption studies become impossible with the accumulation of precipitation on the resin surface. To avoid the precipitation of Ni, Cu, Cd and Zn ions, the adsorption process should be carried out at a pH lower than 6.

**Figure 4:**
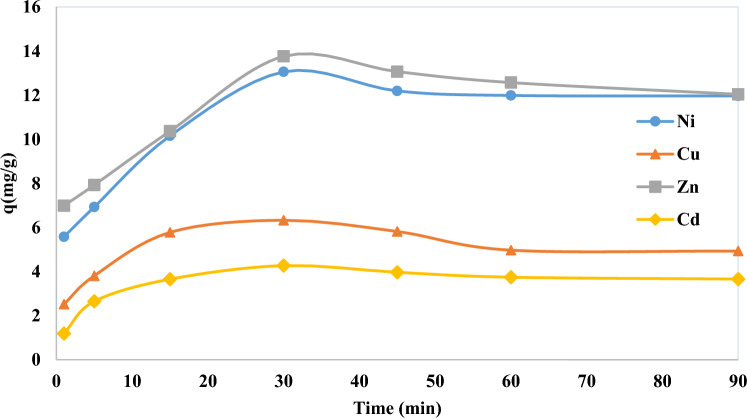
Effect of time on the adsorption of Ni, Cu, Cd and Zn (mg/g) from aqueous solution by CH030 (room temperature, pH 4, 5 g/L of resin).

Figure 4 shows the effect of time on the adsorption of Ni, Cu, Cd and Zn. The time to reach the equilibrium of the two phases is an important parameter for maximum adsorption^81^. The adsorption of the desired ions with 5 g/L of resin from solution at room temperature was fast in the first 30 min because of large number of free surfaces for adsorption and almost the equilibrium was reached for all desired ions after 30 min.

**Figure 5:**
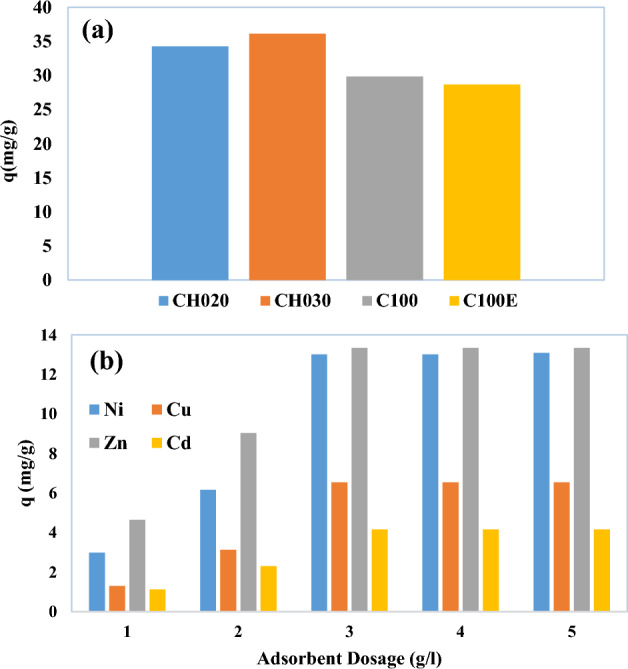
(**a**) Total adsorption of Ni, Cu, Cd and Zn (mg/g) by 5 g/L of CH020, CH030, C100 and C100E (at time 30 min, temperature of 25 C, pH of 4) and (**b**) Effect of resin dosage on the adsorption of Ni, Cu, Cd and Zn (mg/g) by CH030.

The adsorption process on CH020, CH030, C100, C100E resins were carried out at 5 g/L of each resin in leaching solution and pH of 4, temperature of 25 C and time of 30 min. CH030 resin has the highest adsorption of Ni, Cu, Cd and Zn ions due to the presence of amino phosphonic functional groups. After selection of resin type, experiments were conducted to determine the resin dosage. According to Fig. 5a, by increasing the dosage of resin from 1 to 5 g/L, the adsorption of the desired metals increases due to the increase of the contact surface^82^. The adsorption capacity increased rapidly from 1 to 3 g/L and remained almost constant from 3 to 5 g/L.

There are many disturbing ions in the adsorption solution, and a part of the active surface of the adsorbent is filled with these disturbing ions, so the adsorption capacity for the desired ions is reduced.

### RSM results

According to the general definitions in the software manual, models can be considered acceptable if their p-value is less than 0.05 and the lack of fit value of that model is greater than 0.05. According to the analysis of variance (ANOVA) results, single parameters and interactions of the parameters on the model could be explained. ANOVA also contains the sum of squares, degree of freedom (df), mean square, model significant (F) value, and probability (p) value^83,84^. The p-value for both results less than 0.05 is significant, and the lack of fit greater than 0.05 is insignificant^64^. Therefore, the analysis of the variance of the quadratic model is approved. R^2^ values for of Ni, Cu, Cd and Zn are 0.9418, 0.9753, 0.9657 and 0.9189, respectively. The results show that the experimental values are well-fitted to the predicted values. ANOVA quadratic regression model for Ni and Cu recovery are presented in Table 3.

**Table 3:** ANOVA quadratic regression model for Ni, Cu, Cd and Zn adsorption.

Source	Sum of Squares	df	Mean Square	F-value	p-value	
Response 1: Ni						
Model	420.17	19	22.11	31.51	< 0.0001	Significant
Residual	25.97	37	0.7018			
Lack of Fit	18.67	27	0.6916	0.9479	0.5722	Not significant
Response 2: Cu						
Model	133.44	19	7.02	76.87	< 0.0001	Significant
Residual	3.38	37	0.0914			
Lack of Fit	2.06	27	0.0764	0.5796	0.8741	Not significant
Response 3: Cd						
Model	37.33	29	1.29	26.25	< 0.0001	Significant
Residual	1.32	27	0.0490			
Lack of Fit	1.06	18	0.0587	1.98	0.1480	Not significant
Response 4: Zn						
Model	470.73	19	24.78	21.47	< 0.0001	Significant
Residual	41.54	36	1.15			
Lack of Fit	35.76	26	1.38	2.38	0.0766	Not significant

The quadratic regression model equations for Ni, Cu, Cd and Zn adsorption in terms of coded factors are given below as Eqs. 8–11, respectively. This equation should not be used to determine the relative effect of the parameter because the coefficients are scaled to match the units of each parameter in the center of the design space. Equation (8) shows that pH is the most effective parameter on nickel adsorption, and with increasing of pH, the amount of nickel adsorption also increases. From Eqs. 9–11, it is concluded that the amount of resin is the most effective parameter on the adsorption of copper, cadmium and zinc. Of course, for all three desired ions, the amount of adsorption decreases with increasing amount of resin.

8 $$ \begin{aligned} {\text{q}}\left( {{\text{Ni}}} \right) & = 6.03 + 0.7064{\text{A}} + 0.0124{\text{B}} + 2.35{\text{C}} - 1.71{\text{D}} - 0.0188{\text{E}} - 0.0227{\text{AB}} \\ & \quad - 0.3237{\text{AC}} - 0.2945{\text{AD}} - 0.1631{\text{AE}} - 0.0341{\text{BC}} + 0.0470{\text{BD}} - 0.0822{\text{BE}} - 1.22{\text{CD}} \\ & \quad + 0.2748{\text{CE}} + 0.0822{\text{DE}} - 0.3351{\text{A}}^{2} + 0.0780{\text{B}}^{2} + 1.36{\text{C}}^{{2}} + 0.{\text{1371 D}}^{{2}} \\ \end{aligned} $$

9 $$ \begin{aligned} q\left( {Cu} \right) & = 2.83 + 0.1615A + 0.0037B + 0.8300C - 1.39D - 0.0049E - 0.0116AB - 0.1719AC \\ & \quad - 0.1705AD - 0.0077AE - 0.0127BC + 0.0017BD - 0.0208BE - 0.3878CD - 0.0171CE \\ & \quad - 0.0325DE + 0.0974{\text{A}}^{2} + 0.01535{\text{B}}^{{2}} + 0.{\text{6171 C}}^{{2}} + 0.{\text{5793 D}}^{{2}} \\ \end{aligned} $$

10 $$ \begin{aligned} q\left( {Cd} \right) & = 2.84 + 0.3322A + 0.0169B + 0.3040C - 0.5632D - 0.0173E + 0.0927AB - 0.2393AC \\ & \quad - 0.1491AD - 0.1209AE - 0.1336BC + 0.0472BD - 0.0488BE - 0.3219CD + 0.0257CE \\ & \quad - 0.2036DE - 0.1153A^{2} + 0.0185B^{2} + 0.0426C^{2} - 0.0338D^{2} - 0.1138ABE + 0.0497ACE \\ & \quad + 0.0773ADE + 0.1907BCE + 0.0155BDE + 0.0872CDE + 0.0234A^{2} E - 0.0011C^{2} E + 0.1041D^{2} E \\ \end{aligned} $$

11 $$ \begin{aligned} q\left( {Zn} \right) & = 8.44 + 0.8534A + 0.1038B - 0.5268C - 3.24D + 0.4303E - 0.1336AB - 0.4819AC \\ & \quad - 0.4685AD - 0.2131AE - 0.0785BC + 0.0753BD - 0.1776BE - 0.3021CD - 0.1658CE \\ & \quad - 0.3675DE - 0.1835{\text{A}}^{2} + 0.2395{\text{B}}^{2} + 0.0923{\text{C}}^{{2}} + {1}.{81 }\;{\text{D}}^{{2}} \\ \end{aligned} $$

**Figure 6:**
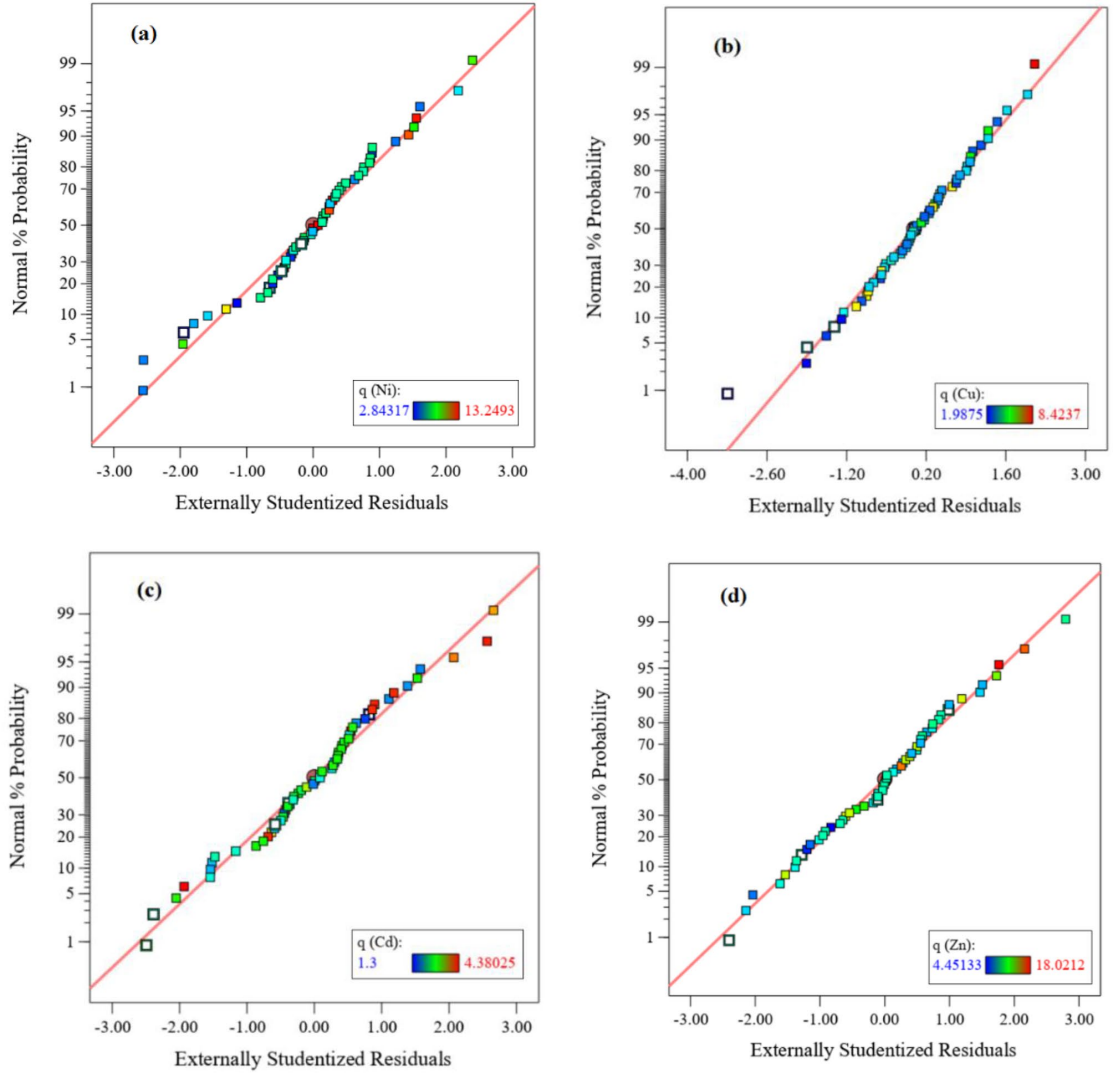
Residual normal probability of (**a**) Ni, (**b**) Cu, (**c**) Cd and (**d**) Zn.

Based on the results, the regression model obtained from CCD is consistent with the experimental values and is used to optimize the experimental parameters. The conditions of data normality for Ni, Cu, Cd and Zn and the dispersion of data to identify with more errors were recognized for model correction. For model validation, the difference between the actual and predicted data is calculated and defined as the residual^85^. According to Fig. 6, the results show that the error rate for all responses is normally distributed and the adequacy of the model is confirmed.

**Figure 7:**
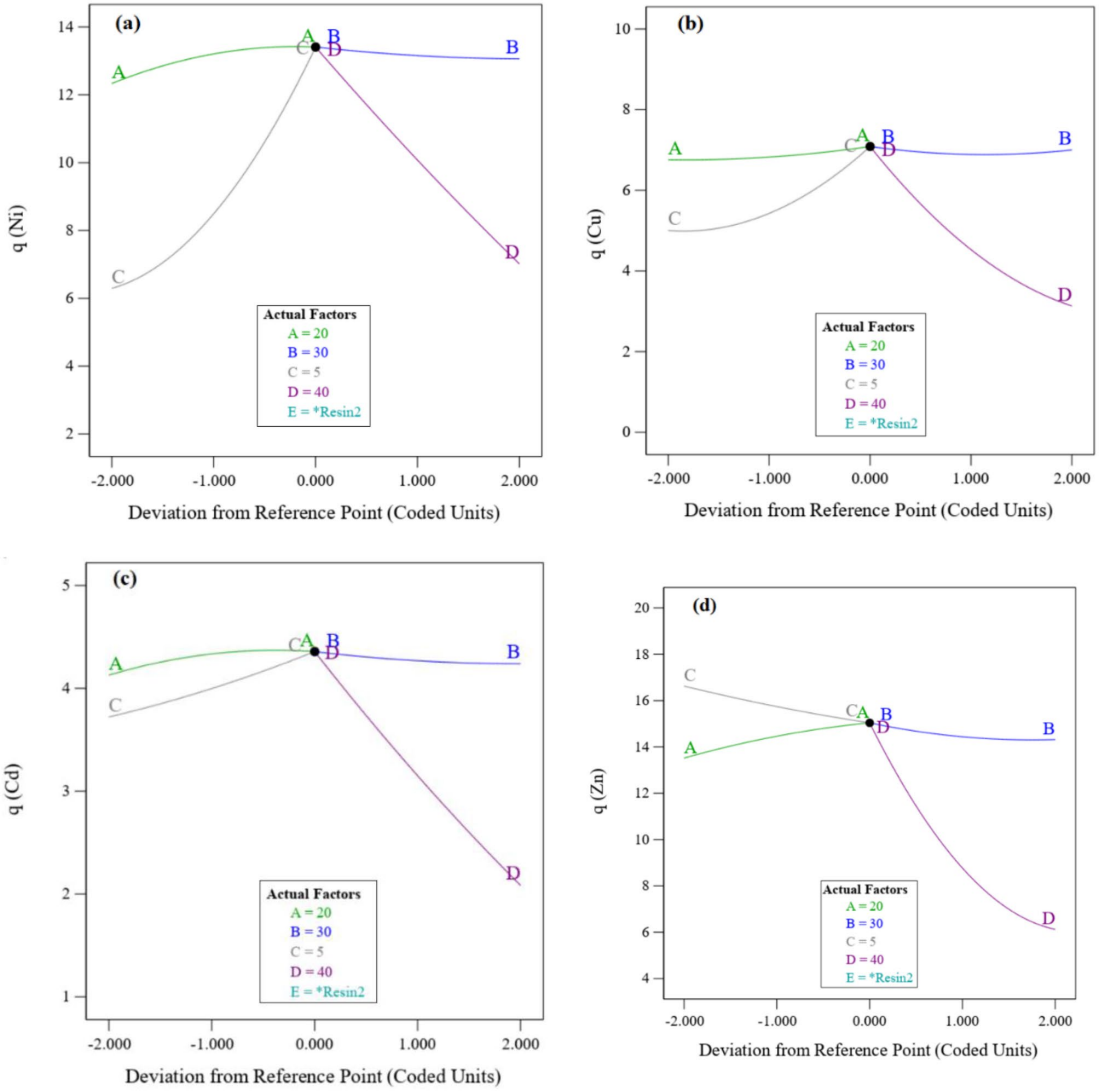
Effect of operating variables on (**a**) Ni, (**b**) Cu, (**c**) Cd and (**d**) Zn adsorption.

Figure 7 shows that the effectiveness of the resin dosage and pH on Ni, Cu, Cd and Zn adsorption^77^. These essential variables in adsorption of desired metals are compatible with the results of the ANOVA model. By increasing the pH, the competition between the H^+^ and the positively charged metal ion at the surface of resin is reduced. Also, with the increase of OH^-^, metal cations have a greater tendency to form complexes and sediments instead of adsorption, and real adsorption studies become impossible with the accumulation of sediment on the resin surface^86^. Based on Fig. 9, increasing the resin dosage has a decreasing effect on the amount of metal adsorption. By increasing the surface area of the resin to the optimum value increases the efficiency of adsorption^82^. But more than optimum point, due to the overlap of the adsorption sites as a result of excessive crowding of the resin particles and also reduction of the contact time required to reach equilibrium, reduces the dosage of adsorption.

The optimal adsorption process was carried out at time of 20 min, temperature of 30 °C, pH of 5 and resin dosage of 2 g/L. In these conditions, the adsorption capacity for nickel, copper, cadmium and zinc were obtained 13.408, 7.087, 4.357 and 15.040 mg/g, respectively.

**Figure 8:**
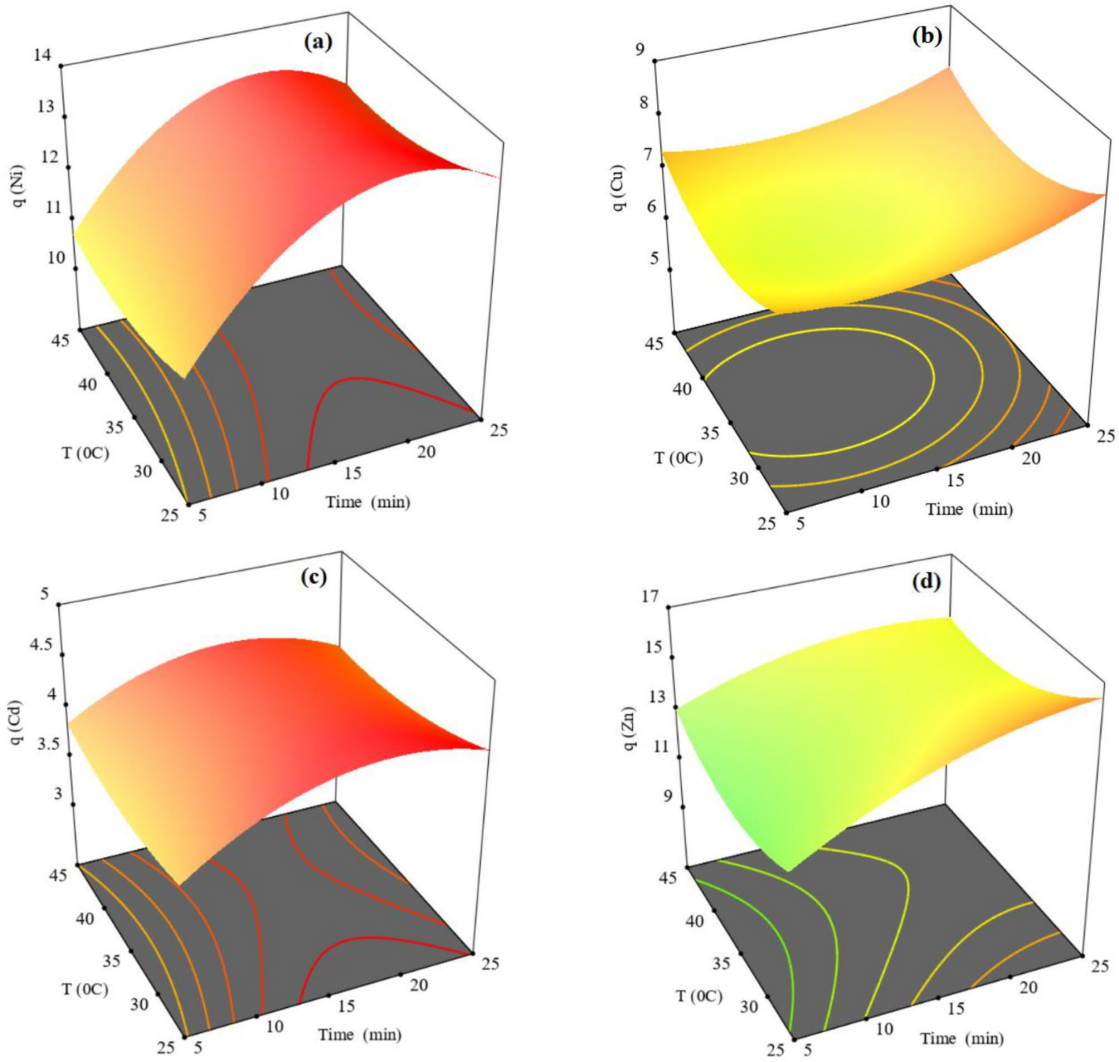
Response surfaces for (**a**) Ni, (**b**) Cu, (**c**) Cd and (**d**) Zn versus time and temperature.

Considering the importance of the simultaneous adsorption of Ni, Cu, Cd and Zn from real leaching solution and evaluation of the interactions of the effective variables in pairs were presented in below. Response surface plots for Ni, Cu, Cd and Zn adsorption versus time and temperature parameters are shown in Fig. 8a–d, respectively. As shown, the highest recovery efficiency is achieved at 30 °C for 20 min. Metal adsorption capacities of the resin increases with increase in adsorption time^77^. The adsorption rate increased sharply in first 20 min because Ni, Cu, Cd and Zn took more chance to be adsorbed by binding sites. Adsorption rate for Ni and Cd decreased than the first 20 min because the majority of resin sites was saturated. But adsorption rate for Cu and Zn after 20 min increases slowly, which these two ions need more time to reach equilibrium conditions. Temperature has not shown much influence upon the adsorption of Ni and Cd. But for Cu and Zn adsorption, the observed decrease in adsorption rate with increasing temperature indicates weak binding interaction between active sites and metal ions, which supports physical adsorption^82^. In addition, physical adsorption reactions are usually exothermic. Hence, the adsorption rate generally increases with decreasing temperature.

**Figure 9:**
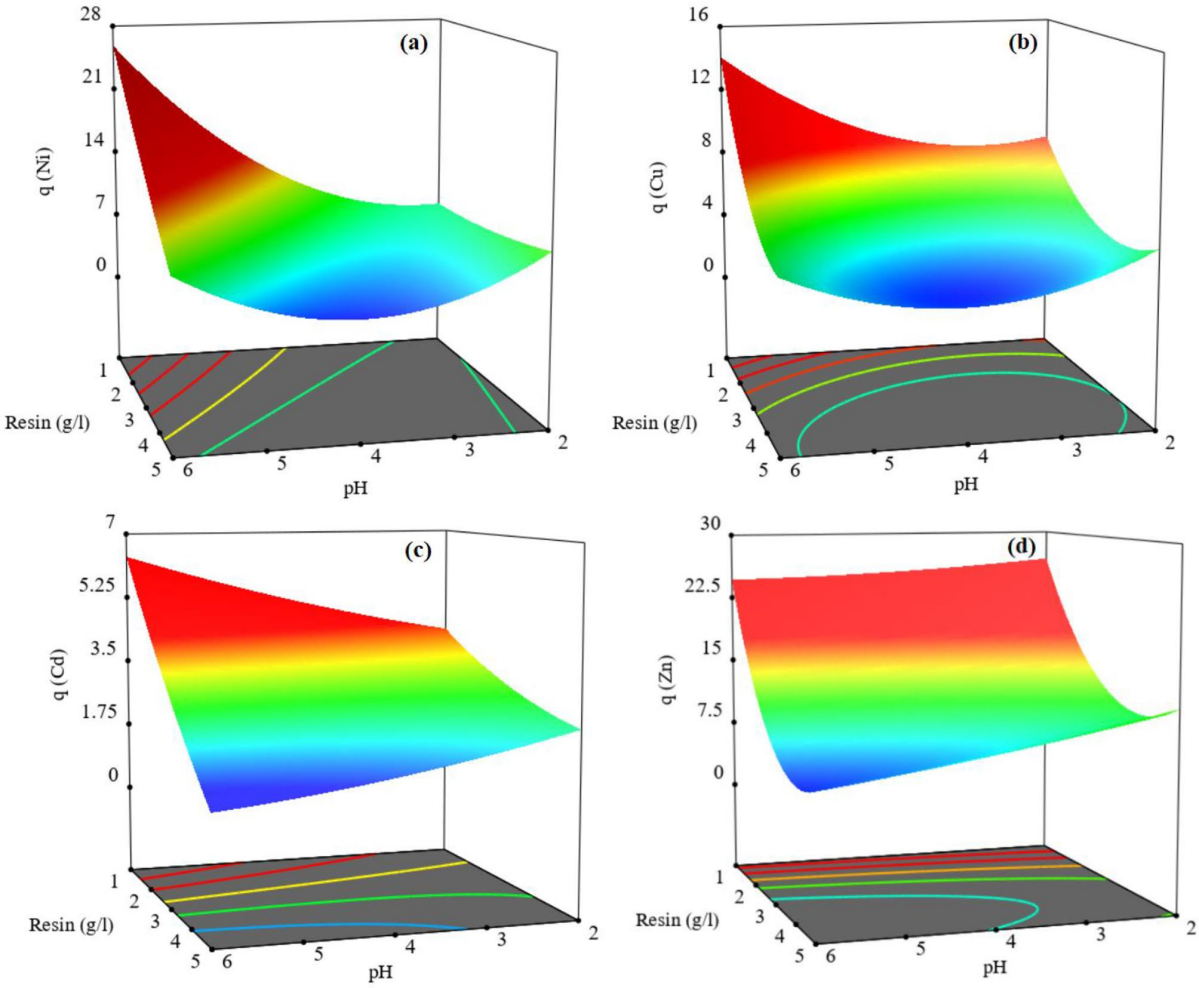
Response surfaces for (**a**) Ni, (**b**) Cu, (**c**) Cd and (**d**) Zn versus pH and resin dosage.

The effect of resin dosage and pH on Ni, Cu, Cd and Zn adsorption is shown in Fig. 9a–d, respectively. By increasing the resin dosage more than 2 g/L and probably overlapping of particles, the contact surface decreases. As the pH increases to 5, the negative charge of the resin surface increases and then adsorption rate increases^81^. But at pH > 5, both the mechanisms of surface adsorption and precipitation of the hydroxide phase have worked in parallel, which leads to an error increase in the dosage of adsorption. Therefore, the optimal pH is 5.

### Adsorption kinetic

**Figure 10:**
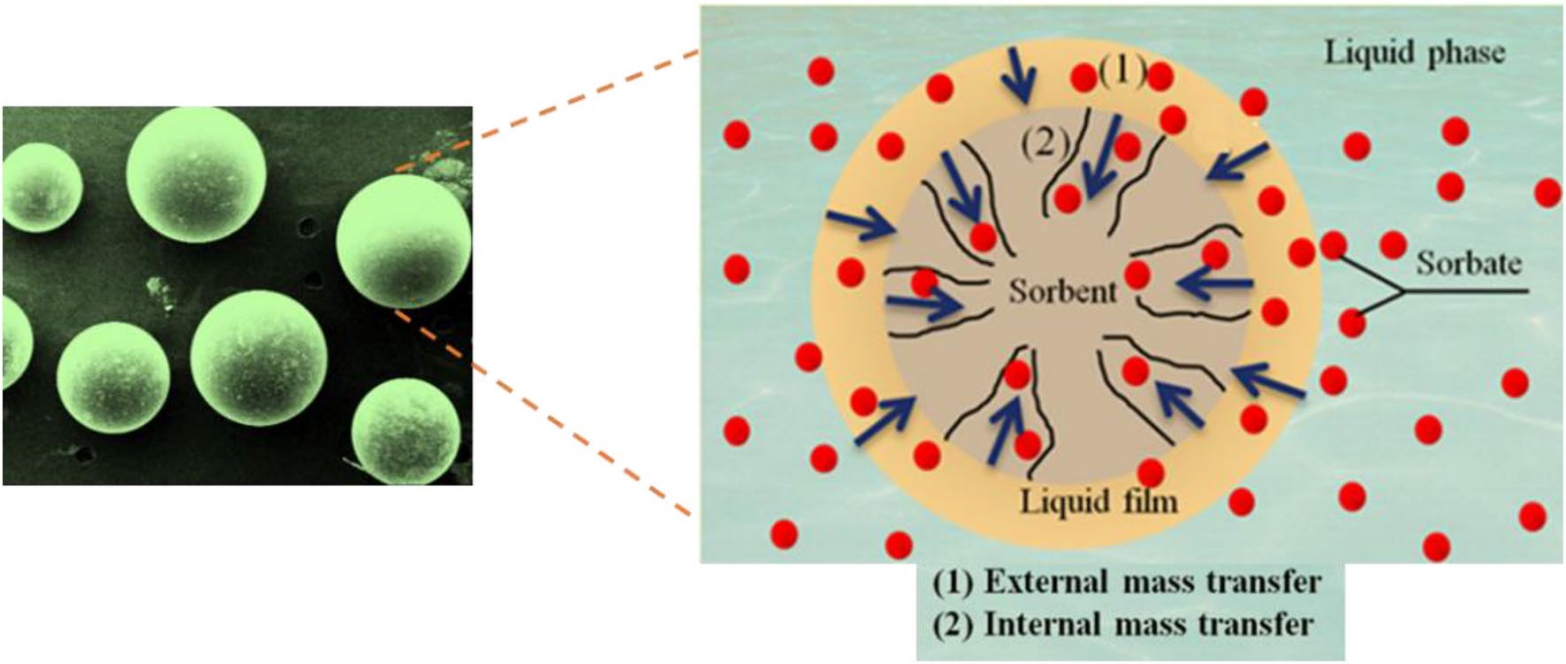
The interaction of functional groups of CH030 with metal cations.

Knowing the adsorption kinetics is essential for the design of adsorption systems. As shown in Fig. 10, in the first stage, the adsorbate is located in the liquid layer around the adsorbent. In the second stage, due to the concentration difference between the surrounding liquid layer and the surface of the adsorbent, mass transfer occurs inside the cavities of the adsorbent. Finally, the adsorbates are adsorbed in the active sites inside the adsorbent.

The pseudo-first-order (PFO) kinetic model accomplished in cases where the initial concentration of the adsorbent is high, or there are few active sites in the adsorbent, or the adsorption is not controlled by the active sites of the adsorbent. Lagergren (1898) firstly proposed the PFO kinetic model by Eq. (12)^69^.

12 $$ q_{t} = q_{e} (1 - {\text{exp}}\left( { - k_{1} t} \right) $$

The pseudo-second-order (PSO) kinetic model is used in conditions of low concentration of adsorbent and material adsorption in active sites of the adsorbent. Ho et al. (1996) firstly used the PSO kinetic model for adsorption of lead onto peat as Eq. (13)^69^.

13 $$ q_{t} = k_{2} q_{e}^{2} t/\left( {1 + k_{2} q_{e} t} \right) $$

where q_e_ and q_t_ are the adsorption capacity per unit mass adsorbent (mg/g) at equilibrium and t time, respectively. The slope of the graph of qe versus time indicates the speed constants (k_1_ and k_2_).

**Figure 11:**
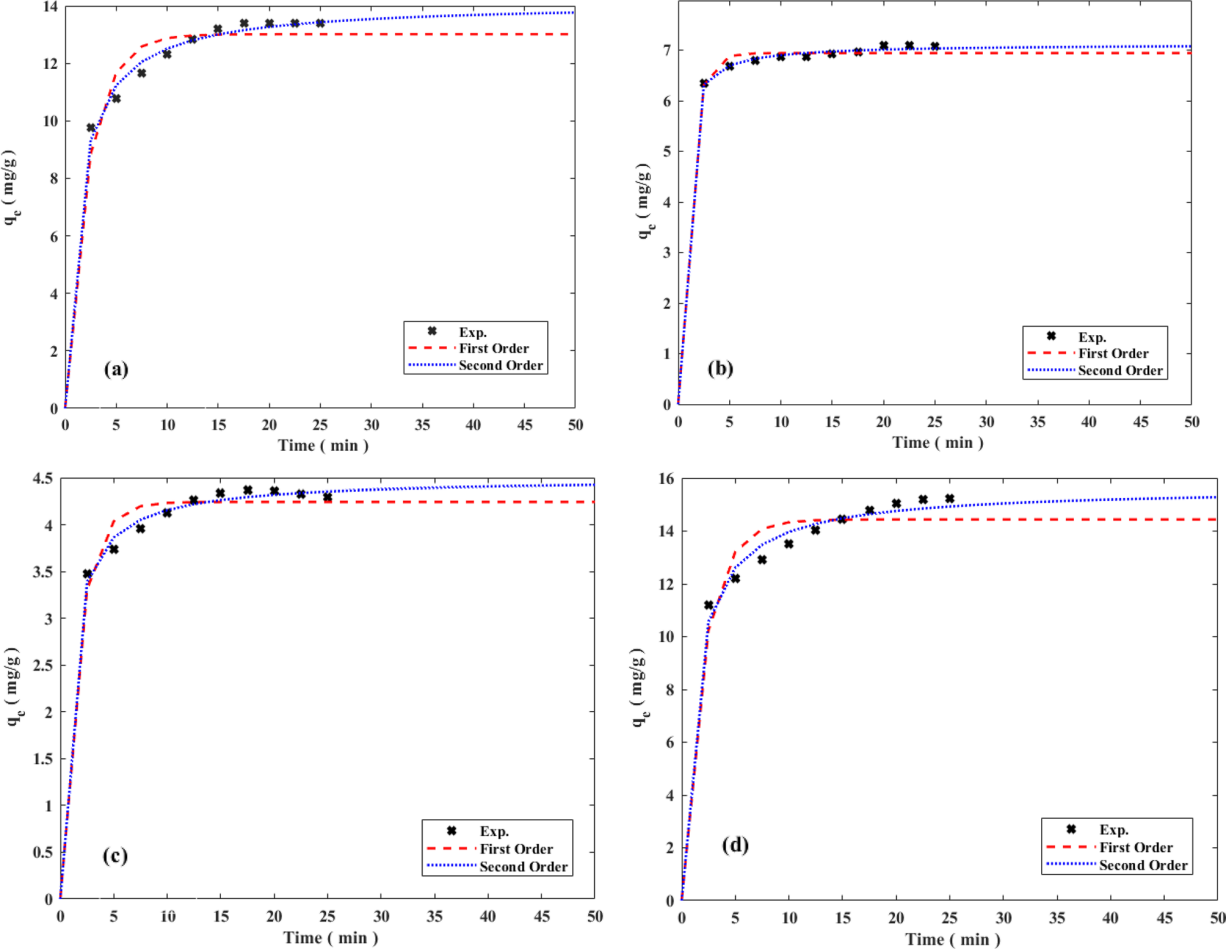
Adsorption kinetic plot for estimation of the parameters for (**a**) Ni, (**b**) Cu, (**c**) Cd and (**d**) Zn.

**Figure 12:**
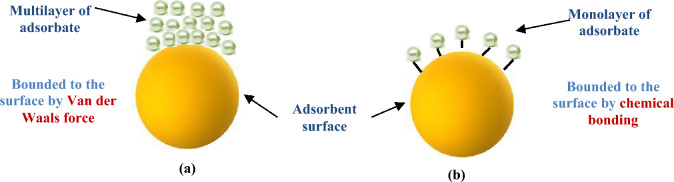
The mechanism of (**a**) physical and (**b**) chemical adsorption.

The adsorption kinetic experiments were carried out to evaluate the rate of nickel, copper, cadmium and zinc adsorption on the CH030. The plot of q_e_ versus time and R^2^ values in both PFO and PSO kinetic models are presented in Fig. 11 and the parameters of the models are shown in Table 3. According to Table 4, R^2^ values for the PSO model are higher than R^2^ values for the PFO model. The experimental data fit better with the PSO model than the PFO model. The parameters in the PSO kinetics indicate that the rate-limiting step may be chemical adsorption related to the adsorption activity or the ion exchange between the adsorbent and the adsorbate. In physical adsorption, the bonding between adsorbate and adsorbent is by weak Van der Waals forces, but the chemical bond is formed between adsorbate and adsorbent surface^70^ (Fig. 12).

**Table 4:** **-** Adsorption kinetic parameters of the Ni, Cu, Cd and Zn.

Kinetic models ions	PFO parameters			PSO parameters		
	q_e_ (mg/g)	Constant (k_1_)	R^2^	q_e_ (mg/g)	Constant (k_2_)	R^2^
Nickel	13.014	0.45522	0.88694	14.121	0.05507	0.97595
Copper	6.941	0.95985	0.84351	7.121	0.44174	0.97157
Cadmium	4.244	0.60581	0.85548	4.496	0.27237	0.96854
Zinc	14.440	0.49352	0.82300	15.650	0.05310	0.95618

### Adsorption thermodynamics

Thermodynamic parameters including free Gibb’s energy (ΔG_0_), enthalpy (ΔH_0_), and entropy (ΔS_0_) can be calculated using the following relations:

14 $$ \Delta G = - RTlnk_{d} $$

15 $$ \Delta G = \Delta H - T\Delta S $$

16 $$ lnK_{d} = - \frac{\Delta H}{{RT}} + \frac{\Delta S}{R} $$

where R is calculated equal to 8.314 J mol^−1^ k^−1^. T is the solution absolute temperature (K) and K_d_ is distribution coefficient. The distribution coefficient is obtained from Eq. (17):

17 $$ k_{d} = \frac{{\left( {C_{0} - C_{e} } \right)}}{{C_{e} }} \times \frac{V}{W} $$

where C_0_ and C_e_ are the initial and equilibrium concentrations in mg L^−1^, V is the volume of the solution in ml and W is the mass of the adsorbent in g^71,72^. For calculating the thermodynamic parameters, the effect of the temperature on the metals adsorption was investigated by time-based analyses, when the adsorption systems were analyzed until adsorbate concentration in the solution became constant (4 g/L).

**Figure 13:**
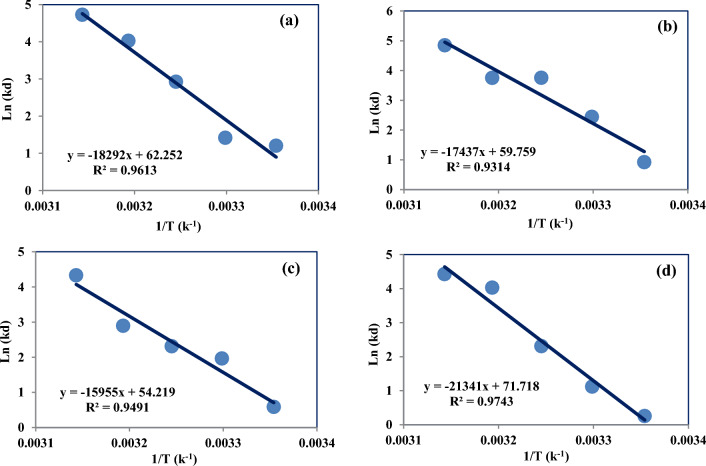
Van't Hoff Plot for estimation of the thermodynamic parameters for (**a**) Ni, (**b**) Cu, (**c**) Cd and (**d**) Zn.

Thermodynamic parameters for the adsorption were calculated from the variations of the thermodynamic equilibrium constant K_d_ that the van't Hoff equation is shown by drawing a linear graph of ln K_d_ versus 1/T at different temperatures based on Eq. (16) for these systems. It is possible to extract the values of adsorption enthalpy and adsorption entropy, respectively, using the slope and intercept of the plots in Fig. 13.

The ΔG values of the adsorbent CH030 at the different experimental temperatures were all negative, and the absolute values of ΔG increased with increasing of temperature (Table 5), which indicated that the adsorptions of nickel, copper, cadmium and zinc were beneficial with the increases of temperature. The ΔH values of CH030 adsorbent were greater than 0 (Table 4), indicating that the adsorption processes were endothermic, and adsorption rate increases with increasing temperature^72,73^. The heat required for only ion exchange in the adsorption process is less than 8.4 kJ/mol^74^ but due to Table 5, ΔH values of four cations adsorption were much bigger than 8.4 kJ/mol that concluded in addition to ion exchange, chemical reaction also happened during the adsorption process. In general, the simultaneous adsorption of nickel, copper, cadmium and zinc on the CH030 resin is very complicated. The ΔS value for nickel, copper, cadmium and zinc was greater than 0 that indicates an increase in the free movement and degree of freedom of four cations in the solution^71,75^ Size of the covalent radius of Zn is smaller than Ni, Cu and Cd, and it is possible to establish more bonds. As a result, irregularity or entropy of Zn increases and based on Eq. (15), enthalpy of Zn also increases more than other ions.

**Table 5:** Thermodynamic parameters of the Ni, Cu, Cd and Zn adsorption on CH030 at different temperatures.

T (◦C)	K_d_	ΔG (kJ/mol)	ΔS (J/mol K)	ΔH (kJ/mol)
Adsorption of nickel				
298.15	3.33	− 2.23	0.52	152.09
303.15	4.12	− 4.82		
308.15	18.62	− 7.41		
313.15	55.95	− 10.00		
318.15	112.86	− 12.58		
Adsorption of copper				
298.15	2.52	− 3.16	0.50	144.98
303.15	11.50	− 5.65		
308.15	42.77	− 8.13		
313.15	42.54	− 10.61		
318.15	127.36	− 13.10		
Adsorption of cadmium				
298.15	1.80	− 1.75	0.45	132.66
303.15	7.11	− 4.00		
308.15	10.08	− 6.26		
313.15	18.05	− 8.51		
318.15	75.86	− 10.77		
Adsorption of zinc				
298.15	1.29	− 0.35	0.60	177.44
303.15	3.06	− 3.33		
308.15	10.04	− 6.31		
313.15	56.01	− 9.29		
318.15	83.46	− 12.27		

## Conclusion

In this work, the optimization of simultaneous adsorption rate of Ni, Cu, Cd and Zn was done by predicting the model behaviour and realizing the effects of the operating parameters on adsorption with RSM. A suitable R^2^ values through RSM-CCD of Ni, Cu, Cd and Zn are 0.9418, 0.9753, 0.9657 and 0.9189, respectively. Increasing the time of the adsorption to 20 min increases the adsorption rate because of the Ni, Cu, Cd and Zn ions have more chance to be adsorbed by active sites. Due to the weak bonds between the metal ions and the active sites of the resin at high temperatures, increasing the temperature does not have a significant effect on the adsorption rate. Increasing the resin dosage up to 2 g/L, which does not cause accumulation of particles, increases adsorption. At pH > 5, surface adsorption and precipitation of the hydroxide phase have happened, which leads to an error increase in the calculation of adsorption rate. The adsorption rate of two resins CH020 and CH030 was compared and more adsorption was done with resin CH030. The priority of metal adsorption in the optimal conditions is: Zn > Ni > Cu > Cd. In addition, Gibbs free energy values and high enthalpy values indicate the chemical interaction between the adsorbent surface and Ni, Cu, Cd and Zn ions in the solution. It is suggested to use the results of batch adsorption in a pilot-scale column in order to industrialize the process. Investigating the process conditions and economic issues is needed to transfer the laboratory results to the industrial scale. Also, comparing the results of this method with other methods can be considered.

## Data Availability

The data used and analyzed during the current study available from the corresponding author on reasonable request.
